# Forage biomass and nutrient quality in brown midrib (BMR) compared to conventional *sorghum*: A meta-analysis approach

**DOI:** 10.5455/javar.2025.l883

**Published:** 2025-03-24

**Authors:** Slamet Widodo, Wijaya Murti Indriatama, Yenny Nur Anggraeny, Mohammad Miftakhus Sholikin, Anuraga Jayanegara, Teguh Wahyono

**Affiliations:** 1 Research Center for Animal Husbandry, National Research and Innovation Agency, Bogor, Indonesia; 2 Research Center for Food Crops, National Research and Innovation Agency, Bogor, Indonesia; 3 Animal Feed and Nutrition Modelling Research Group (AFENUE), IPB University, Bogor, Indonesia; 4 Faculty of Animal Science, IPB University, Bogor, Indonesia; 5 Research Center for Food Technology and Processing, National Research and Innovation Agency of Indonesia, Gunungkidul, Indonesia

**Keywords:** Brown midrib, conventional, forage, meta-analysis, *sorghum*

## Abstract

**Objectives:**

The primary objective of this study is to provide a comprehensive analysis of the differences between conventional and brown midrib (BMR) *sorghum* in terms of biomass, nutrient quality, nutrient digestibility, and animal performance.

**Materials and Methods:**

A comprehensive database was created by integrating 73 datasets from 29 articles. The different studies were denoted as random effects, while the BMR *sorghum* variety was described as a fixed component. Afterward, these two aspects were calculated utilizing a linear mixed model.

**Results:**

According to the findings of the present meta-analysis, conventional *sorghum* produces higher quantities of forage and grain biomass in comparison to BMR (*p* < 0.001). As expected, BMR *sorghum* has a lower content of neutral detergent fiber (NDF), acid detergent fiber, and lignin compared to conventional *sorghum* (*p *< 0.001). The digestibility of BMR *sorghum* performed better than conventional *sorghum* (*p* < 0.001), especially for NDF digestibility (54.98% *vs.* 47.37%). BMR shows suitability as a fodder option for dairy cows due to its superior milk yield compared to conventional *sorghum* (15.04 *vs.* 14.06 kg/day; *p* < 0.01).

**Conclusion:**

In conclusion, BMR *sorghum* produces higher biomass compared to conventional *sorghum*. Nevertheless, in terms of nutrient quality and digestibility, BMR *sorghum* is the most optimal choice. The results will significantly improve animal performance.

## Introduction

*Sorghum* may contribute to the mitigation of climate change and the adaptation to global warming due to various factors: 1) it shows drought resistance; 2) it captures carbon from the atmosphere using the C_4_ photosynthetic pathway; and 3) it has relatively low requirements for water and fertilizer. *Sorghum* (*Sorghum bicolor* [L.] Moench), a type of C_4_ grass, exhibits robust growth during the summer months in temperate climates, but it maintains particular significance in tropical semiarid and arid areas across the world [1,2]. *Sorghum* is a worldwide important crop that is appropriate to many agricultural and environment-related conditions, especially in regions with low rainfall or limited availability of irrigation water [3]. *Sorghum* presents a favorable alternative with potentially lower adoption risks for farmers due to its currently well-established commercial production and supply pathways [4]. *Sorghum* plants have been utilized as alternative fodder in an extensive forage program to optimize land utilization, mitigate drought and crop risks, and fulfill the total fodder requirements of the livestock herd [5]. Besides being applied for fresh feeding, *sorghum* plants are a beneficial, nutritious, and quick-growing fodder that may also be used for hay and silage [6]. According to the presented facts, *sorghum* crops are currently and will continue to be an essential fodder component for ruminant livestock.

*Sorghum* is typically categorized into three groups based on the color of the midrib on its leaves: green midrib, white midrib, and brown midrib (BMR) [7,8]. The induction and description of BMR mutants in *sorghum* were started by Grover et al. [9]. The reason behind the change of BMR color from its normal green or white form remains unknown. However, this transformation could potentially be explained by the synthesis of hydroxy cinnamaldehyde into lignin polymers [10,11]. The BMR mutants, which have been obtained either spontaneously or through chemical mutagenesis, have significantly contributed to the identification of a unique collection of genes whose products are essential for cell wall lignification [2,12]. The lignin concentration of the cell walls is decreased in *Sorghum* BMR mutants, which show yellow to brown midribs. Because they improve the foliage's digestibility for cattle, these improvements are valuable [1,13], as demonstrated both in *sorghum* and Sudan grass, as well as their hybrid varieties [14]. BMR plants have attracted attention as potential feedstocks for biorefining processes because of their lowered lignin content, which enables easier breakdown and conversion [15].

Comparative investigations of phenotypic traits between BMR *sorghum* and the wild type have been frequently reported [7,8,11,16–21]. However, to the best of our knowledge, there has never been a meta-analysis study comparing conventional *sorghum* with BMR *sorghum* on the allocation of biomass production and nutrients. To inform the public, we believe a quantitative review study is necessary to assess the advantages of BMR traits over other *sorghum* varieties. Recent studies [2,9,10] have primarily employed genetic approaches to elucidate the mechanisms controlling lignification in BMR cell walls. In contrast, our study utilizes a meta-analysis approach, synthesizing data from previous research. Therefore, the main purpose of our study was to conduct a comprehensive investigation of the differences between conventional and BMR *sorghum* regarding biomass, nutrient quality, nutrient digestibility, and animal performance.

## Materials and Methods

### Ethical approval

Ethical clearance was not required for the present meta-analysis study.

### Search for keywords

The literature search was conducted using Harzing's Publish or Perish 8th version (Windows GUI Edition). The keywords employed were *sorghum*, brown midrib, and forage. A total of 208 publications were published between 2010 and 2024, collected from PubMed^®^, Scopus^®^, and Google Scholar^®^. The selected literature exclusively consisted of scientific articles/journals.

### Criteria for selection

**Figure 1.**
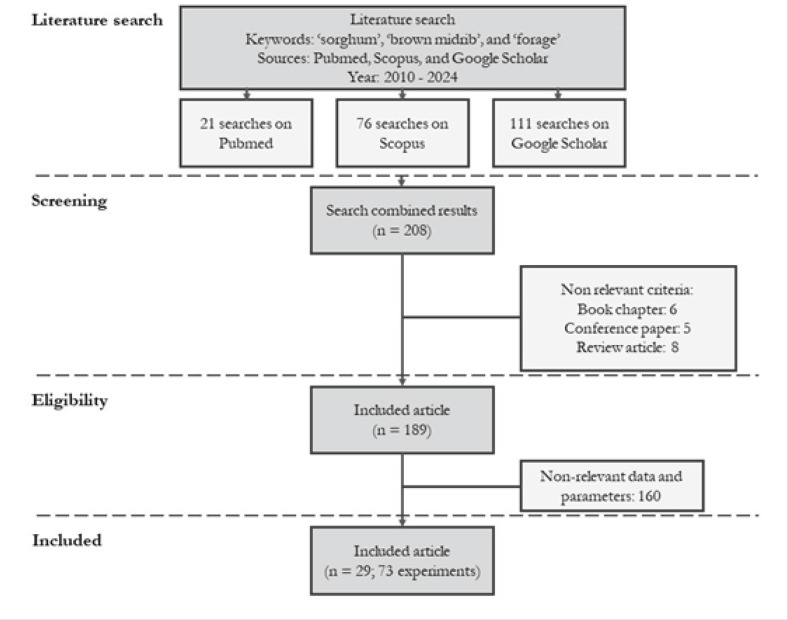
The procedure of selecting and examining articles follows the PRISMA guideline.

The article selection approach adhered to the Preferred Reporting Items for Systematic Reviews and Meta-Analyses (PRISMA) guidelines [22]. The criteria for selection were as follows: (1) The article has been published in a scientific paper. (2) The article was an investigation based on experimental work. (3) The study includes a comparison between BMR and conventional *sorghum*. (4) The subjects of this study were *sorghum*, sudangrass, and *sorghum* x sudangrass. (5) Manuscripts that contain details on harvest age are preferred. Figure 1 displays a graphic illustrating the process of literature selection. Following a comprehensive examination of the entire article, a total of 29 publications, which included 73 experiments, were included in the database and are listed in Table 1.

### Included data

Specific information on the type of *sorghum*, the stage/day of harvesting, the season, and the soil type can be found in Table 1. These details will be further elaborated extensively in the results and discussion section. The parameters compiled include biomass, nutrient quality, digestibility, and performance in animals. Biomass parameters were forage biomass, grain biomass, and plant height. Moisture, crude protein, neutral detergent fiber, acid detergent fiber, lignin, and total digestible nutrient value were the nutrient quality parameters. The digestibilities assessed were dry matter digestibility, organic matter digestibility, neutral detergent fiber digestibility, and *in vitro* true digestibility (IVTD). Average daily gain (ADG), dry matter intake, and milk yield were the animal performance parameters.

### Modeling and statistical analysis

A linear mixed-model methodology was applied for performing statistical meta-analysis on the datasets, which had compatible measurement units [23,24]. The various studies were categorized as random effects, whereas the BMR *sorghum* variety was classified as a fixed component. The statistical analyses were performed using R software version 4.1.2 developed by the R Core Team (http://www.r-project.org/index.html) [25], and the lme4 library version1.1–35.3 (https://cran.r-project.org/web/packages/lme4/index.html). An evaluation and statistical test were performed on the model. The significance of the results was assessed through a one-way analysis of variance. A *p*-value qualifies as significant if it is less than 0.05 (*p *< 0.05), and it tends to be significant if it ranges between 0.05 and 0.1.

## Results

### The characteristics of the data utilized in meta-analysis studies

Table 1 presents the characteristics of research integrated into the meta-analysis study. This research has summarized a total of 29 studies. Together with *sorghum*, we performed a comparative analysis of BMR and non-BMR types in sudangrass [26] and *sorghum* x sudangrass [7,21,27–29]. The cultivars mentioned in the research differ based on the specific local varieties and mutant variants originating from the country in question. The majority of studies did not provide a detailed report on the type of BMR. However, bmr2, bmr3, bmr6, and bmr12 types have been reported by several compiled studies [16,30–32].

The phrase "conventional *sorghum*" is initially used to refer to non-BMR *sorghum*. Therefore, the classification of "conventional" *sorghum* includes many types, such as grain *sorghum*, forage *sorghum*, sweet *sorghum*, and staygreen *sorghum*. The reported stages of harvest also vary, particularly including flowering, soft dough, milk dough, hard dough, mid dough, joint, head, and grain maturity. Due to the various sources of the summarized study, there is also a variation in the reported soil types. Primarily consisting of latosol, vertisol, sand, silt loam, black soil, and clay. *Sorghum* plants are adaptable to various soil structures, altitudes, and climates [33]. This statement is further supported by the variations in growing seasons reported in this meta-analysis (drought, spring, summer, rain, and dry winter).

The data distribution from this meta-analysis study is presented in Table 2. The mean forage and grain biomass were 15.40 and 7.74 Mg/ha dry matter (in DM), respectively. The reported plant height varies between 73 and 311 cm. The crude protein, NDF, ADF, and lignin content of *sorghum* varies significantly due to differences in harvesting ages. The TDN content of *sorghum* forage varies between 44.30% and 61.00%. The digestibility of DM, OM, and NDF showed varying ranges of 39.95%–61.29%, 35.18%–62.00%, and 44.30%–61.00%, respectively, due to variations in nutritional content. There is a lack of studies that show differences in animal performance between BMR and non-BMR treatment. However, it is capable of presenting the ADG, DMI, and milk production variables.

**Table 1.** Summary of research integrated in meta-analysis.

Study no.	Reference	*Sorghum*/ sudangrass	Cultivar name	Harvesting stage/day	Soil type	Season
1	Erbretta et al. [38]	*Sorghum*	No information	No information	No information	No information
2	Wahyono et al. [11]	*Sorghum*	BMR (GH2.3); C (Super-1)	Hard dough	latosol	Drought
3	Pupo et al. [21]	*Sorghum*; *Sorghum* *x* *Sudangrass*	No information	Soft dough	Arredondo-Gainesville (sand and loamy sand)	Spring; summer
4	Ferreira et al. [20]	*Sorghum*	BMR (ADV F7232); C (AF 8301)	Milk-soft dough	Hayter and silt loam	No information
5	Suhartanto et al. [56]	*Sorghum*	BMR (no information); C (Super-2)	No information	No information	No information
6	Sriagtula et al. [19]	*Sorghum*	BMR (Patir 3.7); C (Patir 3.1)	Flowering, soft dough, hard dough	No information	Rain
7	He et al. [67]	*Sorghum*	BMR (Big Kahuna, Big Dragon, Prolific Graze-BMR, Late Graze); C (Jackpot 2180, Monster, Jackpot 1180, Jackpot 4180, Jackpot 1230, Jackpot 3180, Superdry, )	Hard dough	Black soil	Drought
8	Dey et al. [58]	*Sorghum*	BMR (SPV-2018, SPV-2017); C (SSG-59-3, CSV-32F)	No information	No information	No information
9	Harmon et al. [29]	*Sorghum* x Sudangrass	BMR (Honey Graze); C (Sugargrazer)	No information	No information	Spring
10	Ordoñes et al. [42]	*Sorghum*	BMR (SM350); C (DK67)	Milk-dough	crumbly sand, sandy clay loam and sandy loam	Rain
11	Wahyono et al. [50]	*Sorghum*	BMR (G5); C (Numbu)	Flowering, soft dough, hard dough	Latosol	Drought
12	Ferreira et al. [55]	*Sorghum* x Sudangrass	BMR (BR007 x Tx2784bmr, Tx635 x Tx2785bmr); C (BRS 801, Tx636 x Tx2785)	51 day	No information	Dry winter
13	McCuistion et al. [28]	*Sorghum*, *Sorghum* x Sudangrass	BMR (Later Grazer BMR, Pacesetter BMR); C (Later Grazer, Pacesetter)	Mid dough	Clareville clay loam	Drought
14	Vinutha et al. [68]	*Sorghum*	BMR (N610); C (CO 30, COS 28)	80 and 160 day	Medium-fertility vertisol	Rain
15	Scully et al. [30]	*Sorghum*	BMR (bmr6-23); C (BTx623)	139 day	mix (peat, moss, vermiculite, perlite, sand)	No information
16	Sriagtula et al. [41]	*Sorghum*	BMR (Patir 3.7); C (Patir 3.1)	Flowering, soft dough, hard dough	No information	Rain
17	Telleng et al. [69]	*Sorghum*	BMR (Patir 3.7); C (CTY)	Flowering	No information	Rain
18	Yerka et al. [59]	*Sorghum*	BMR (bmr-12); C (A Wheatland × R Tx430)	60 day	Sharpsburg silty clay loam soil	Rain
19	Puteri et al. [18]	*Sorghum*	BMR (Patir 3.7); C (Samurai 1)	Flowering, soft dough, hard dough	No information	No information
20	Li et al. [7]	*Sorghum*, *Sorghum* x Sudangrass	No information	Joint, head, flowering, milk stage, dough stage	Silty loam	No information
21	Li et al. [32]	*Sorghum*	BMR (bmr-12); C (N-12)	86 day	Silty loam	Drought
22	De Aguilar et al. [48]	*Sorghum*	BMR (CMSXS156AxTX2784bmr, CMSXS156AxTX2785bmr, BR001AXTX2784bmr); C (CMSXS156AxTX2784, CMSXS156AxTX2785, BR001AxTX2784)	97 day	Dystrophic Red typical cerrado phase	Dry winter
23	Astigarraga et al. [27]	*Sorghum* *x Sudangrass*	BMR (Candy Graze); C (Supergauchazo)	No information	No information	Spring
24	Portillo et al. [70]	*Sorghum*	BMR (CI0947bmr); C (Sureno)	Grain maturity	No information	Summer
25	Bean et al. [17]	*Sorghum*	No information	Soft dough	Pullman silty clay loam	Rain
26	Beck et al. [26]	*Sudangrass*	BMR (Hayking); C (Piper)	Hard dough	Una silty clay loam	Rain
27	Rao et al. [31]	*Sorghum*	BMR (IS 21887, IS21889, IS21549); C (RSSV 9, ICSV 93046, IS11861)	Flowering	No information	Rain
28	Marsalis et al. [34]	*Sorghum*	BMR (Dairy Master BMR); C (FS-5)	Soft dough	Olton clay loam	Rain
29	Vietor et al. [16]	*Sorghum*	BMR (09248bmr); C (DK52)	After dough	Miller Clay (fine, mixed, thermic vertic haplustolls)	No information

C=Conventional.

### BMR vs. conventional sorghum: Biomass comparison

Forage and grain biomass in conventional *sorghum* was higher than in BMR *sorghum* (Table 3; *p* < 0.001). There was approximately a 14.33% decrease in the forage biomass of BMR. The average value of forage biomass obtained in DM yield was 13.47 BMR versus 15.40 Mg/ha (conventional). Related to biomass value, conventional *sorghum* also had a higher plant height than BMR (224.83 *vs.* 197.11 cm; *p* < 0.05). The high amount of forage and grain biomass in conventional *sorghum* can be attributed to the wide range of cultivars present, which include varieties of *sorghum* specifically developed for grain, fodder, and silage production.

### BMR vs. conventional sorghum: nutrient comparison

The nutritional composition of plants utilized for animal fodder plays an essential role in influencing the growth, reproduction, and production of cattle [34]. Table 3 presents a comparison of the nutritional composition of BMR and conventional *sorghum* forages. The moisture content of BMR *sorghum* forages tends to be higher compared to that of conventional cultivars (76.25% *vs.* 75.61% DM; *p* = 0.099). The protein content of BMR *sorghum* was significantly greater than conventional cultivars (*p* < 0.005). Based on the original claim of the BMR type, it has been demonstrated that the fiber content (NDF, ADF, and lignin) of BMR forage is significantly lower than conventional *sorghum* (*p* < 0.001). The TDN estimate for forage *sorghum* was greater than non-BMR, resulting in a value of 56.63% compared to 54.29% (*p* < 0.001).

### BMR vs. conventional sorghum: digestibility and animal performance comparison

The digestibility of BMR *sorghum* forage is superior to that of conventional *sorghum* across all parameters (*p* < 0.05; Table 3). In addition to *in vivo* digestibility data, the IVTD parameters also indicate that BMR *sorghum* forage is more easily digested than conventional *sorghum* (*p* < 0.001). Regarding animal performance, despite the small sample size (*n* < 10), there is evidence indicating that the BMR *sorghum* leads to a better milk yield compared to conventional *sorghum* (*p* < 0.01; *n* = 5). Ruminants that receive BMR *sorghum* also tend to improve weight gain (*p* = 0.071).

*Biomass, nutrient quality, and nutrient digestibility of BMR and conventional sorghum: effects of harvesting stage*

Additionally, we want to present information on the impact of harvest age on biomass output, nutritional quality, and digestibility for *sorghum* forages (both BMR and conventional). These results will be shown in Table 4, along with the meta-data results. We compare the following three physiological phases of harvest: flowering, soft dough, and grain maturity. The following three harvest phases are dominant in the 29 articles that have been collected for discussion. Regarding grain biomass and plant height, harvest phases did not differ significantly. The soft dough phase showed the greatest forage biomass, averaging 17.08 Mg/ha (in DM yield). The flowering phase produces the lowest quantity of forage (9.46 Mg/ha). The crude protein content exhibited a decreasing pattern as the maturity phase increased (*p* < 0.001). The harvest phase of soft dough generated the lowest percentage of fiber fractions (NDF and ADF) (*p* < 0.001). Furthermore, compared to the other two harvest phases, the soft dough phase tends to have the lowest amount of lignin (*p* = 0.076). Notably, there were no significant differences observed in the digestibility and TDN values.

**Table 2.** Distribution of data in meta-analysis study.

No	Variable	Unit	*n*	Mean	SD	Upper	Lower
Biomass							
	Forage	DM Mg/ha	42	15.40	7.19	32.40	3.01
	Grain	DM Mg/ha	10	4.74	2.58	7.57	0.52
	Plant height	cm	17	224.83	54.01	311.00	73.00
Nutrient quality							
	Moisture	%	24	75.61	10.35	88.56	43.18
	Crude protein	% DM	58	8.41	2.99	16.45	3.16
	Neutral detergent Fiber	% DM	53	59.39	8.19	82.50	43.90
	Acid detergent fiber	% DM	45	36.47	6.20	58.60	24.70
	Lignin	% DM	36	5.33	1.65	9.65	2.90
	Total digestible nutrient	% DM	16	54.29	4.86	61.00	44.30
Digestibility							
	Dry matter	%	12	53.48	7.11	61.29	39.95
	Organic matter	%	10	50.13	8.43	62.00	35.18
	Neutral detergent fiber	%	20	47.37	9.01	65.10	23.60
	IVTD	%	10	54.76	20.63	78.40	18.89
Animal performance							
	Average daily dain	kg/day	3	0.72	0.23	0.86	0.45
	Dry matter intake	kg/day	6	17.86	10.28	24.50	1.68
	Milk yield	kg/day	5	14.06	2.99	19.10	11.64

DM = dry matter, IVTD = *in vitro* dry matter digestibility.

## Discussion

The key attributes of *sorghum* BMR have been described in the studies conducted by Scully et al. [30] and Rao et al. [31]. Mutations in the bmr6 allele result in a 15%–50% reduction in cinnamyl alcohol dehydrogenase (CAD) activity compared to normal, leading to a reduction in lignin levels. The bmr12 allele decreases the abilities of the lignin biosynthesis enzyme caffeic acid O-methyltransferase (COMT), which breaks down lignin H and G. The genetic changes in BMR *sorghum* are believed to directly impact the amount of forage and grain biomass. The decrease in crop yield is believed to be associated with the properties of BMR in plants [35,36]. However, additional molecular investigations are required to investigate these phenomena. Joy et al. [37] demonstrated a positive correlation between stover yield and the lignin content in various *sorghum* varieties. This finding presents evidence that BMR *sorghum* produces biomass at lower levels than conventional *sorghum*. The physiological reasoning behind this phenomenon is that *sorghum* straw's total biomass will decrease as the lignin content in the stem decreases. This is because the level of lignin was positively correlated with stem (structural) biomass density, specifically in internode parenchyma [38]. The genetic changes in BMR *sorghum* are expected to enhance the plant's ability to be converted into ethanol but will considerably decrease its biomass yield [10]. Da Silva et al. [12] showed that a 12% decrease in mean biomass yield was observed for BMR lines in comparison to the isogenic lines. Bean et al. [17] also reported that when comparing the biomass production of the forage *sorghum* (FS) and forage *sorghum*-BMR (FS-BMR) classes over multiple years, it was shown that BMR cultivars exhibited a 12.5% reduction compared to non-BMR cultivars.

According to a study summarized by Beck et al. [26], hybrid BMR *sorghum* showed a reduction of 12%–15% in DM yield as compared to non-BMR *sorghum*. The BMR mutation consistently reduced the amount of biomass allocated to the stems of photoperiod sensitivity *sorghum*. Additionally, the introduction of these characteristics significantly reduced the panicles-to-shoot ratio of photoperiod-insensitive *sorghum* [39]. In this meta-analysis, it was found that BMR *sorghum* has a decreased biomass yield compared to conventional *sorghum*. However, the total biomass remains higher than that of maize and millet, regardless of using the same maintenance strategy [40].

**Table 3.** Meta-analysis results for biomass, nutrient quality, nutrient digestibility, and animal performance (BMR *vs.* conventional *sorghum*).

No	Variable	Unit	*n*	BMR	Conventional	*p*-value
Biomass						
	Forage	DM Mg/ha	42	13.47	15.40	0.001
	Grain	DM Mg/ha	10	2.59	4.74	0.001
	Plant height	cm	17	197.11	224.83	0.024
Nutrient quality						
	Moisture	%	24	76.25	75.61	0.099
	Crude protein	% DM	58	8.91	8.43	0.029
	Neutral detergent fiber	% DM	53	57.50	59.45	0.001
	Acid detergent fiber	% DM	45	33.67	36.51	0.001
	Lignin	% DM	36	3.77	5.36	0.001
	Total digestible nutrient	% DM	16	56.63	54.29	0.001
Digestibility						
	Dry matter	%	12	63.29	53.48	0.002
	Organic matter	%	10	56.21	50.13	0.031
	Neutral detergent fiber	%	20	54.98	47.37	0.001
	IVTD	%	10	63.14	54.76	0.001
Animal performance						
	Average daily gain	kg/day	3	0.84	0.72	0.071
	Dry matter intake	kg/day	6	17.53	17.86	0.399
	Milk yield	kg/day	5	15.05	14.06	0.004

DM = dry matter, IVTD = *in vitro* dry matter digestibility.

Although decreased levels of lignin have been linked to lower yields, genetic predisposition, and environmental conditions may significantly influence actual yield [17]. The low forage biomass in BMR *sorghum* is hypothesized to be linked to the plant's natural self-defense mechanism. The low lignin content in BMR *sorghum* can affect plant stability when there is a high quantity of forage and grain yield. Lignin is crucial for providing structural stability at the organ level, as significant reductions in lignin content led to plants being unable to maintain a standing position [41]. Based on our investigation of multiple studies, most of them indicate that BMR-type *sorghum* generally has a lower biomass production compared to conventional *sorghum* [11,17,18,26,27,34,42,43]. The present meta-analysis's findings can be applied as evidence that the forage biomass factor must be considered for attention while developing BMR characteristics. In many scenarios, lignin can be lowered without affecting yield or fitness. Various variables and genetic background play a crucial role [44]. Consistent with studies on the application of *sorghum* for energy, the *sorghum* hybrids' high yield, in combination with their improved chemical characteristics, will be beneficial in determining which components are most suitable for the development of next-generation biofuels [45].

It is necessary to determine the moisture content of *sorghum* forage due to its frequent application as a silage material. Several variables, especially the type of forage, temperatures, and moisture levels, influence the successful fermentation of silage [46]. According to Bean et al. [17], for optimal silage quality, the moisture content of the total plant should range between 65% and 70% at the time of harvest. The average moisture content for BMR and non-BMR *sorghum* forages in our compilation study was 76.25% and 75.61%, respectively. Applying wilting treatment [47] is necessary to enhance the forage quality of BMR and conventional *sorghum* when used as silage material. In addition to planting season and *sorghum* variety, the harvesting stage [48] and plant density [42] have a significant impact on the moisture content of *sorghum* forage.

**Table 4:** Meta-analysis results for biomass, nutrient quality, and nutrient digestibility: effects of harvesting stage.

No	Variable	Unit	Flowering	Soft dough	Grain maturity	*p*-value
Biomass						
	Forage	DM Mg/ha	9.46	17.08	12.43	0.004
	Grain	DM Mg/ha	0.38	4.16	5.11	0.420
	Plant height	cm	198.62	216.15	233.50	0.403
Nutrient quality						
	Crude protein	% DM	9.26	7.7	5.5	0.001
	Neutral detergent fiber	% DM	62.99	51.66	57.5	0.001
	Acid detergent fiber	% DM	40.04	29.24	33.5	0.001
	Lignin	% DM	5.22	4.56	7	0.076
	Total digestible nutrient	% DM	46.08	55.57	57.5	0.304
Digestibility						
	Dry matter	%	62.94	56.88	60.5	0.912

The lower crude protein value of conventional *sorghum* forage in comparison to the BMR type could be attributed to its greater biomass value. According to Billman et al. [49], the quality of forage with high biomass will be reduced since it has a relatively low crude protein content. However, Bleier et al. [13] found it challenging to identify consistent effects of cultivars on protein levels, unlike most variables associated with growth. This limitation could be attributed to the fact that mutations in the BMR type were exclusively examined at specific lignin concentrations. The changes in specific nutritional levels, such as protein, might be attributed to the indirect impact of a relative decrease in lignin concentration. Nevertheless, further investigation is required. Reducing lignin levels in the BMR type affects the availability of energy from carbohydrates in *sorghum* plants [50]. There is a positive correlation between crude protein content and the energy content of forages [6]. However, the protein content in forage is highly influenced by land management practices [45]. Feeding diets with higher levels of starch may enhance the utilization of protein from the BMR *sorghum* [5].

The present findings of a meta-analysis confirm that the BMR cultivars of *sorghum* have a lower lignin content compared to conventional *sorghum*. In addition, BMR *sorghum* has been found to have low levels of NDF and ADF, which are fiber fractions commonly linked to lignin. In general, reducing the amount of lignin decreased the levels of ADF and NDF while simultaneously improving digestibility [8]. Various previous studies have elucidated the mechanism of how the *sorghum* BMR gene promotes lignin reduction. The bmr2 gene is responsible for encoding methylenetetrahydrofolate reductase, whereas the bmr4 and bmr19 genes encode folylpolyglutamate synthase. Both enzymes play a role in catalyzing processes involved in the synthesis of S-adenosyl methionine [2,10,32]. The mutations in bmr6 result in a lack of CAD activity, whereas the mutations in bmr12 result in a drop of COMT activity [15,51]. The bmr-18 genotype carries a non-functional allele, bmr18, which is characterized by the presence of a termination codon in the first exon of the COMT gene due to a G to A replacement [52]. The bmr30 mutant carries a change in a phenylpropanoid biosynthetic gene, which plays a crucial role in the interaction between flavonoids and monolignols. Both flavonoids and monolignols are essential for the biosynthesis of lignin in plants [2].

Our findings additionally confirm the correlation between reduced yields of biomass in the BMR type and reduced amounts of fiber (NDF and ADF) and lignin. The fresh biomass yield and the *sorghum*'s fiber content (hemicellulose, cellulose, and NDF) have been found to have negative correlations, indicating that the characteristics may be related [44]. Significant associations were observed between biomass yield attributes and lignin content. Therefore, choosing a higher biomass production would increase plant lignin content, consequently reducing digestibility [35]. Reducing the amount of lignin increases the availability of structural carbohydrates, such as cellulose and hemicellulose, to enzymatic hydrolysis, a process that converts them into sugars that can be fermented [53]. Mutations in the BMR gene have been observed to enhance the efficiency of converting biomass by optimizing the amount of soluble carbohydrates in plant material [38]. The findings of this study further confirm the statement made by Yang et al. [4], who showed that BMR *sorghum* varieties had lesser lignin content, making them beneficial for animal forage markets despite their generally lower yields.

The differences in mean nutritional contents between BMR and conventional *sorghum* significantly affect the estimation of TDN value. It is possible to precisely estimate the TDN content of forage using nutrient composition data [54]. Our compiled investigations demonstrate variations in calculations, with the majority of them depending on NDF and CP as the fundamental factors for the calculations. The greater TDN value in BMR-type *sorghum* is influenced by the lowered NDF level [21,26,55] and increased crude protein content [18,19,56]. The TDN concentration is positively correlated with higher nutrient content, particularly crude protein, and negatively correlated with the fiber components, specifically ADF, NDF, and lignin [19].

The superior digestibility of *sorghum* forage BMR, as measured by its dry matter, organic matter, and NDF content, is consistent with its high TDN value and low fiber components (NDF, ADF, and lignin). The BMR trait enhances bioenergy production by raising both the concentration of soluble sugars in the stem and its digestibility [38]. Low levels of fiber fraction will increase energy conversion from feed, thereby increasing dry matter, organic matter, and NDF digestibility [50]. Enhanced digestibility of the NDF component has a beneficial impact on energy availability and digestive function, possibly leading to increased dry matter intake [17]. Low lignin content may enhance the digestibility of cell walls, thereby increasing cattle's energy intake [8]. On the contrary, a high quantity of lignin in the cell wall is unfavorable since it inhibits microbial digestion in the rumen, resulting in reduced digestibility of forage cell walls [39].

BMR characteristics modify lignin metabolism in plants, resulting in decreased lignin levels. This can enhance the digestion of fiber and increase animal performance when compared to conventional cultivars [13,17,27]. Dey et al. [57] also reported that the organic matter digestibility and metabolizable energy values for BMR *sorghum* were significantly greater (*p* < 0.001) compared to both conventional and sweet *sorghum* stovers. Regarding the *in vitro* assay, the IVTD showed a strong negative correlation (*r* ≤ −0.72) with ADF, NDF, and lignin, with little differences observed among the three [17]. Wahyono et al. [11] showed that there were weak negative correlations between IVTD and the NDF and ADF content, with *R*² values of −0.201 and −0.202, respectively. Low lignin properties in the BMR type increase the probability of hemicellulose breakdown, hence accelerating the fermentation process and leading to higher *in vitro* digestibility [58].

Animal performance, as measured by daily gain and milk yield, has a positive association with the digestibility and high nutrient value of BMR *sorghum* (Table 3). The BMR gene and variations in the nutritional value of forage linked to digestibility may be the cause of the ADG trends [29]. However, it is important to note that the dry matter intake compared to conventional *sorghum* remains identical. These findings demonstrate that BMR *sorghum* has a greater energy conversion rate for its nutrients compared to conventional *sorghum*. The financial benefits and animal performance of livestock-fed BMR *sorghum* were greater than those of wild-type *sorghum* [59]. The increased NDF digestibility in the BMR forage *sorghum*s may result in greater milk output compared to conventional *sorghum* [60]. A higher carbohydrate concentration in the diet increases the energy density of BMR forage *sorghum*, which is correlated with increased milk production [21]. According to previous meta-analyses [61,62], BMR *sorghum* is commonly used as a silage material and is nutritionally balanced with corn silage for improving dairy cow milk production. Forage *sorghum* genotypes with BMR have higher fiber digestibility and may produce milk yields comparable to corn silage [5].

According to our meta-analysis, the best period to collect *sorghum* forage is at the soft dough stage. This ensures that the lignin content is minimal and the biomass is sufficiently high. This is appropriate regardless of the *sorghum* type, whether it is BMR or non-BMR. Puteri et al. [18] also reported that BMR and conventional types of *sorghum* produce high biomass at the harvest age of 95 days after sowing (DAS), or the soft dough phase. In the grain maturity phase, many leaves may experience senescence, which will reduce biomass production. Our findings are in line with the findings of Suradiradja et al. [63], who employed a Decision Tree Algorithm. Previous research has determined that the optimal age for harvesting *sorghum* is approximately 84 DAS when the plant achieves a height of a minimum of 138.5 cm.

Lyons et al. [64] also reported that harvesting before the soft dough stage leads to higher NDF digestibility, ADF, and CP contents, while reducing non-fiber carbohydrate, starch, and DM concentrations. In our findings, CP content decreases as the generative phase increases. During the panicle emergence stage to the physiologic maturity stage, crude protein concentration continuously decreases [65]. This result follows the findings of Wahyono et al. [50], who reported that there is an inhibition mechanism in protein synthesis when entering the mature period. The low fiber fraction content in the soft dough phase will naturally increase the easily digestible energy content. The *sorghum* crops produced the highest mean juice ethanol yield at the soft dough stage, which was followed by the hard dough stage with a slight difference [66].

Despite our efforts to provide a comprehensive analysis, we recognize some limitations in our study. Our literature search was limited to PubMed^®^, Scopus^®^, and Google Scholar^®^, excluding Web of Science^®^ due to access restrictions. Additionally, the sample size for animal performance parameters (average daily gain, dry matter intake, and milk yield) was small due to the limited number of sources. Finally, our discussion on the effects of the harvesting stage is restricted to the flowering, soft dough, and grain maturity phases.

## Conclusion

The present meta-analysis study indicated that conventional *sorghum* has higher amounts of forage and grain yield, although it contains inferior nutrient content and digestibility in comparison to BMR *sorghum*. According to the findings, BMR reveals lower fiber and lignin levels compared to conventional *sorghum*. The high digestibility of DM and fiber is affected by the above facts. Further investigation is required to fully understand the impact of BMR *sorghum*-based feed on daily weight gain and milk production, considering the current number of studies is limited. More research needs to be done to find ways to increase biomass and grain yield while keeping the fiber content, which is a big benefit of BMR *sorghum* forage crops.

## List of abbreviations

ADF, acid detergent fiber; ADG, average daily gain; BMR, brown midrib; C, conventional; CAD, cinnamyl alcohol dehydrogenase; COMT, caffeate O-methyltransferase; CP, crude protein; DM, dry matter; DAS, days after sowing; DMI, dry matter intake; ha, hectare; IVTD, in vitro true digestibility; Mg, mega gram; NDF, neutral detergent fiber; OM, organic matter; SD, standard deviation; TDN, total digestible nutrient.
